# CASSIOPE: An expert system for conserved regions searches

**DOI:** 10.1186/1471-2105-10-284

**Published:** 2009-09-10

**Authors:** Virginie Lopez Rascol, Anthony Levasseur, Olivier Chabrol, Simona Grusea, Philippe Gouret, Etienne GJ Danchin, Pierre Pontarotti

**Affiliations:** 1 EBM UMR 6632.LATP, 3 place V. Hugo - 13 331 Marseille cedex 03 France; 2 UMR-1163, INRA de Biotechnologie des Champignons Filamenteux, IFR86-BAIM. ESIL, 163 avenue de Luminy, CP 925, 13288 Marseille Cedex 09, France; 3 Universités Aix-Marseille I & II, UMR-1163, case 925, 163 avenue de Luminy, 13288 Marseille Cedex 09, France; 4 UMR 1301, INRA, F-06903 Sophia-Antipolis, France; 5 UMR 6243, CNRS, F-06903 Sophia-Antipolis, France; 6 UMR 1301, UNSA, F-06903 Sophia-Antipolis, France

## Abstract

**Background:**

Understanding genome evolution provides insight into biological mechanisms. For many years comparative genomics and analysis of conserved chromosomal regions have helped to unravel the mechanisms involved in genome evolution and their implications for the study of biological systems. Detection of conserved regions (descending from a common ancestor) not only helps clarify genome evolution but also makes it possible to identify quantitative trait loci (QTLs) and investigate gene function.

The identification and comparison of conserved regions on a genome scale is computationally intensive, making process automation essential. Three key requirements are necessary: consideration of phylogeny to identify orthologs between multiple species, frequent updating of the annotation and panel of compared genomes and computation of statistical tests to assess the significance of identified conserved gene clusters.

**Results:**

We developed a modular system superimposed on a multi-agent framework, called CASSIOPE (Clever Agent System for Synteny Inheritance and Other Phenomena in Evolution). CASSIOPE automatically identifies statistically significant conserved regions between multiple genomes based on automated phylogenies and statistical testing. Conserved regions were searched for in 19 species and 1,561 hits were found. To our knowledge, CASSIOPE is the first system to date that integrates evolutionary biology-based concepts and fulfills all three key requirements stated above. All results are available at

**Conclusion:**

CASSIOPE makes it possible to study conserved regions from a chosen query genetic region and to infer conserved gene clusters based on phylogenies and statistical tests assessing the significance of these conserved regions.

**Source code **is freely available, please contact: Pierre.pontarotti@univ-provence.fr

## Background

Comparative genomics and the reconstruction of ancestral genomes provide landmarks better understand the biological rules governing evolution. The most obvious way to make progress in ancestral genome reconstruction is to compare the organizational structure of conserved genomic regions in a large number of informative species [1]. Hypotheses can then be formulated to account for such conserved genomic regions:

• Observed conserved regions are due to chance and are not biologically significant.

• Conserved regions result from a common ancestral region through inheritance.

• Conserved regions are due to evolutionary convergence with possible selective pressure.

CASSIOPE is able to reject the null hypothesis (conserved regions due to chance) in favor of one of the two alternatives, but cannot distinguish between them (ancestral regions or convergence). In literature reports, conserved regions are frequently defined through BLAST [2] or alignment by similarity search to determine putative "orthologous" genes [3,4]. Furthermore, the significance of the observed conserved gene clustering has to be statistically tested to reduce the risk of false positives. Several tools and databases (Phig's [5]), Ensembl [6]) provide information on conserved regions across different species but even when they do use phylogenetic methods, there is no statistical processing assessing the significance of the conserved regions.

In contrast, a few methods seek conserved regions using statistics [7,8] but do not offer a phylogeny-based distinction between orthologs and paralogs. Thus, in [8], GRIMM-synteny computes conserved regions based on gene markers ("orthologous" sequences or "orthologous" alignments that users have to input) and a distance threshold.

There are two requirements for identifying biologically significant conserved regions:

• Identification of conserved markers (orthologs or paralogs between different species)

• Identification of significantly conserved clusters of these markers

Currently, there is no software available that detects conserved regions by providing a phylogenetic determination of conserved markers together with and a score for their significance. Furthermore, those resources that are available pre-compute conserved regions on a limited number of species, eliminating the possibility or running searches using custom-input regions.

In short, biologists today find themselves needing one set of tools to identify orthologous or paralogous markers and then another set of tools to evaluate the significance of observed conserved regions. The lack of software able to provide automated output of statistically-estimated information on conserved regions at several-genome scale together with the growing amount of genomic data being filed prompted us to automate comparative analysis based on conserved genes clusters, through the CASSIOPE project.

The CASSIOPE project proposes new methodology using evolutionary biology-based concepts. First, orthologs and paralogs are detected via phylogenetic analysis. Several approaches not based on phylogenetic analysis claim to find orthology. However, the clustering requires a complete genome and, in the case of lineage-specific differential paralog loss, provides spurious data that contradict the identification of orthologs and paralogs based on phylogeny. Secondly, chromosomal regions from different species that are inherited from a common ancestor have a higher probability of containing homologs than under neutrality. This probability has to be rigorously calculated to give a score on the evolutionary history of the species compared. These evolutionary concepts have been embedded in CASSIOPE.

CASSIOPE deploys the following core technologies:

• Data-processing system: the computer system is a modular system with several agents (virtual machines that work on specific tasks) deployed in conjunction with an expert system that communicates with every agent and takes rule-based decisions to answer initial biological questions. The rulesets of the expert system can be updated, removed or added, just as a human scientist would.

• Data flexibility: searches can be run for newly-sequenced regions or genomes. The comparative data is initially pooled and computed, and then recomputed when saved data is older than one month.

• Detection of orthologous genes by robust phylogenetic reconstruction.

• Statistical score to assess significance of conserved regions.

• Reverse-search feature making it possible to extend the initial searches.

The stability and reliability of CASSIOPE were determined using several tests on large genomic regions (containing several hundred genes).

## Methods

As previously stated, the search for biologically-relevant conserved genomic regions requires phylogenetic orthology assessment, statistical testing, and as many available genomes as possible. The breakthrough offered by CASSIOPE is that it integrates all three of these key steps in a single automated process:

• Phylogeny: orthologous/paralogous genes are determined by phylogenetic methods (using Figenix, [9]). Phylogenetic information also allows reconstruction of the evolutionary history, and therefore more accurate ancestral genome reconstruction.

• Statistical test: we apply a statistical test for each conserved region to assess the significance of the observed conserved gene clusters [for a detailed description see [10]], taking multigenic families and paralogs into account.

• Reverse search: when conserved regions between a region A1 of species A and a region B1 of species B are found, the reverse conserved regions are subsequently checked, i.e. after finding conserved regions from A1 to B1, a reverse search is performed from B1 in order to screen for the conserved region in species A. This step will either find part of the same region A1 or expand this region or a different new region (whether or not it is on the same chromosome). This reciprocal process thereby finds multi-directional conserved regions from all species to all species (all-against-all), enables to confirm conserved regions, boundary these regions, and highlight translocations, duplications and other evolutionary phenomena at chromosome level.

### Orthologous conserved regions

Here we describe the algorithm used in CASSIOPE with, as starting material, specific genomic region Ra in species A located on chromosome Ca:

(1) For each gene from Ra, we search for orthologous genes in multiple species using phylogenetic methods.

If {Ga_1_,..., Ga_*n*_} are genes of Ra, then for each Ga_*i *_1 <*i *<*n*, we obtain:

{Os_1_,..., Os_*p*_} a list of orthologous genes of gene *i *in several species s_*j *_1 <*j *<*p*

(2) Once phylogenetic reconstruction of all genes from this region Ra has allowed the determination of orthologous genes, we classify them as a function of

i. species

ii. chromosome

iii. number of orthologs on this chromosome ≥ 3.

Thus, several clusters in different species can be obtained.

(3) We complete all clusters by including the non-orthologous genes found inside clusters delimited in step (2).

(4) We assess statistical support for conserved regions between start region and each new region found in previous steps using a "binomial test" [10]





We test the null hypothesis H_0 _and the alternative hypothesis H_1_:

H_0_: Conserved genes clustering between start region and identified end region results from a random process.

H_1_: Conserved genes clustering between start region and orthologous end region does not result from a random process.

*p*: probability of observing a number *k *of orthologous genes (randomly pulled out of *n *genes) all mapped to the same region. Therefore, *p *is the probability that each gene contained in the start region will have one (or several) orthologs in the end region.

*q*: probability that orthologs are localized elsewhere in the target genome (*q *= 1-*p*).

If Ra is start region and Rb a putative "target" conserved region found in species B,

then:

*p *= number of genes in Rb/genes in genome B.

*n *= number of genes in species A which have at least one orthologous gene in species B (found by phylogeny)

*k *= number of genes in Rb that are orthologous to genes in Ra.

If a given gene from Ra has multiple orthologous genes in species B (due to duplication after speciation of A and B), the probability of finding an orthologous gene in Rb is higher than for one-to-one orthologous genes. Consequently, in the case of multiple orthologs, *k *is corrected as follows:

If g_*i*_, 1 <*i *<*n *is a gene in Ra

And if *l*_*i *_= number of orthologous genes (found from g_*i*_) in the species B genome

And if *m*_*i *_= number of othologous genes (found from g_*i*_) that fall in region Rb

Then



(5) Reverse search: we restart the algorithm with all the newly found regions as starting regions using the following logical rules: unless the new region is included in or overlaps a region that has been already studied, we restart with this new region.

The algorithm stops when all the conserved sites have been calculated.

### Paralogous conserved regions

CASSIOPE is also able to identify paralogous conserved regions (conserved genes clusters in the same genome) exactly in the same manner as it finds orthologous conserved regions.

Working with a fixed time-window on duplication events and with phylogenetic trees, paralogous genes are detected by finding matching duplication nodes, i.e. nodes that correspond to the defined time-window. For instance, the search for paralogous genes that appear after duplication of vertebrate nodes eliminates duplication nodes that contain species other than vertebrates.

Paralogous genes are then clustered and "non-matching" clusters (less than three genes) are removed.

For each cluster, conserved regions are computed. The statistical test is the same as for orthologous regions.





Where: *p *= number of genes in Rb/genes in B genome

*n *= number of genes in species A that are at least one paralog in species B (found by phylogeny)

*k *= number of paralogous genes in Rb

## Results and Discussion

### System

**Figure 1:**
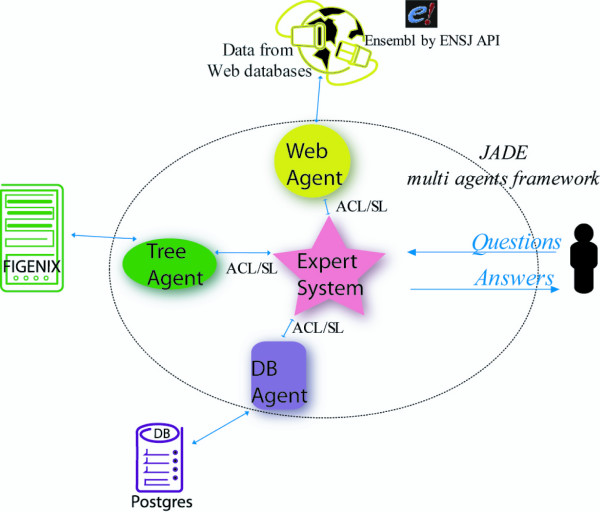
**CASSIOPE multi-agent system, showing all the agents and the communications (blue) between them, together with non-system elements**. Pink: Expert System; Violet: persistence agent and Postgres database; Green: tree agent and FIGENIX platform; Yellow: Web agent.

**Figure 2:**
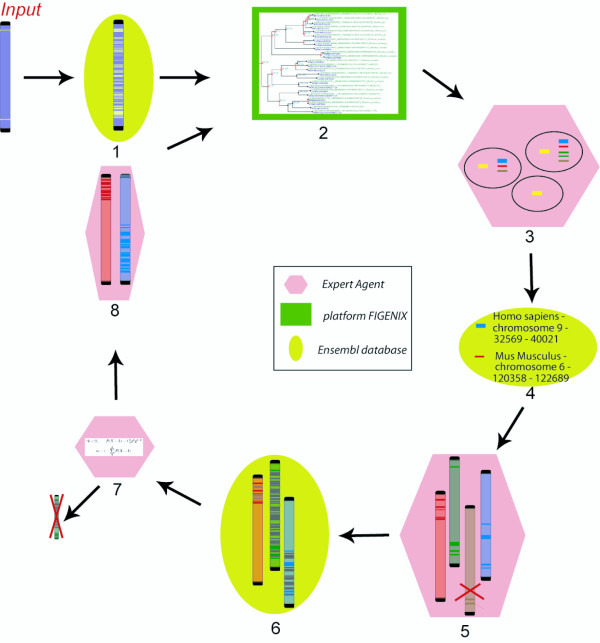
**The diagram depicts how processes and agents are overlaid**. Agents are pink, green and yellow, as represented in Fig 1. Arrows represent communication channels between the expert system and the other agents. The persistence agent is not represented as it is used throughout the process: (input). The user gives the two boundary genes of the target region - (1) The region is completed by all genes present in the Ensembl database - (2) The phylogenetic tree is computed for each gene - (3) Orthologous genes are calculated (forester) - (4) Each orthologous gene is located on the chromosome in its species - (5) Orthologous genes are clusterized if they are in the same chromosome and the same species. If the cluster contains fewer than three genes, it is removed - (6) Clusters are completed with genes contained in the region and that are not orthologous genes - (7) A score is calculated for each cluster: if the conserved site is not significant, then the cluster is removed - (8) The system restarts with new regions.

Expansion of the panel of genomes available for comparison will allow us to construct higher resolution models of genome evolution. However, the vast amounts of data involved make it impossible to manually identify all conserved regions among a large number of species. Another key requirement is regular updates on conserved region data, as genome assembly and annotation can be refined over time as new genomes are released. CASSIOPE is a modular system superimposed on a multi-agent framework. The expert system controls three slaves. This is a centralized system where one of the "agents" has a global view of the process and drives the non-intelligent slaves (Figure 1). Each virtual machine has a specific task and all the machines work together to address user queries. The process developed in CASSIOPE (Figure 2) involves several tasks, such as phylogenetic reconstruction or consulting web databases, and each task is independent from the others. The expert system contains a full ruleset allowing decision-taking on information received. The whole process is contained in the expert system and uses other agents to obtain the information required.

### a) Agents

#### Expert System

This is the core of CASSIOPE. The Expert System communicates with different agents to answer the following question: which genomic regions are significantly conserved? It receives queries and tries to find the required information in database. If information is incomplete, unavailable or outdated (> 1 month), the system deduces the questions it has to ask to the other agents.

One example rule, taken from reverse search mode, concerns when to restart with new region R.

If the question for R has already been solved:

R is included in another solved region

or R overlaps another solved region

then the Expert System does not restart with R

else it restarts with R.

Each time it registers conserved regions, an e-mail is sent to the user indicating the species-pairs involved and the associated significance score.

At the same time, the expert system creates reports for each cluster found, indicating the different steps performed and the elements used for deductions.

#### Tree Agent

CASSIOPE uses the FIGENIX computer platform [9] to assess phylogenetic relationships (orthology/paralogy). FIGENIX allows phylogenetic trees to be built via specific pipelines. In CASSIOPE, the CassiopePhyloM pipeline is used to reconstruct the desired trees. From the query sequence, a dataset of putative homologous sequences is first constructed using BLASTp using protein sequences from ENSEMBL. CASSIOPE filters the raw dataset to eliminate potentially non-homologous sequences (E-value threshold: 10^-4^), disturbing alignments, and duplicates. The next step uses CLUSTALW to produce an alignment that is then modified to eliminate large gaps. Since phylogenetic analysis is achieved at domain level, we next detect these domains with HMMPFAM. For each domain alignment (extracted from the original alignment), a bias correction phase is run to eliminate: *i*) non-monophyletic "repeats", *ii*) sequences with divergent composition, which is done using the amino-acid composition test in TREE-PUZZLE software (with an alpha risk set to 5%), and *iii*) sites not under neutral evolution SHIFT-FINDER. Indeed phylogenetic reconstruction methods are not tolerant to sites highly divergent to neutral evolution and molecular clock. Sites not respecting this rule potentially produce errors in trees' reconstruction and thus have to be masked. Once the domains have been "purified", and after congruent domain selection using the HOMPART test in the PAUP package, a new alignment is built by merging preserved parts of the domains' alignments. This new alignment is then used to generate three phylogenetic trees using NJ, ML (with TREE-PUZZLE) and MP (with PAUP package) methods. By comparing the topologies of these trees with the PSCORE command ("Templeton winning sites" test) from the PAUP package and the KISHINO-HASEGAWA test from the TREE-PUZZLE package, these trees are fusioned to produce a unique consensus tree. This consensus protein tree can then be compared with a reference species tree (the tree of life from NCBI) to deduce proteins orthologous to the query sequence [see Additional file 1].

FIGENIX is accessible online ; however, the Tree Agent runs tasks directly by querying FIGENIX without interface.

#### Database Agent

All features are registered in a local database (managed with postgresql ). Each time the Expert System searches for information, it asks the Database Agent whether this information already exists and whether it is out of date, i.e. > 30 days old.

For instance, when phylogenic trees are screened for a given gene G, the database agent checks whether G is already included in orthologs list of another gene, taking the FIGENIX task identifier and asking FIGENIX to send the corresponding gene tree (this avoids re-computing existing trees). This database can also be consulted using a "telescope viewer" interface (see section 3.2) that allows user-friendly visualization of results.

#### Web Agent

We have developed an agent that searches for information on remote sources on the Web. As a source of genomic and proteomic sequence data, we chose the Ensembl [6] database as it is a non-redundant and frequently updated database allowing retrieval of gene locations on different chromosomes at the base pair (bp) level, and that can be accessed via an API from a JAVA library (ENSJ).

### b) Telescope viewer

CASSIOPE enables users to view all the conserved sites registered in the database. Whenever CASSIOPE receives new conserved region queries, the results are saved and made visible using the telescope viewer.

This viewer has three levels:

• Chromosome level: for a given species, a chromosome can be chosen showing regions that are conserved in other species and their identifiers.

• Region level: by clicking on "chromosome region" in the last level, a region on the chromosome appears in front of boxes. Each box represents conserved regions on each chromosome of each species. It allows a global view of species sharing the conserved regions and of their distribution in each species.

• Gene level: by clicking on a box in other species, conserved sites appear between two regions with all the genes of the two regions together with homologous between-gene links, making it possible to explore gene distribution in both regions. Ensembl link and FIGENIX task identifier are available for each gene.

### c) CASSIOPE Parameters

CASSIOPE allows users to modify several of the parameters used at different steps in order to fine-tune the software:

#### CASSIOPE. global parameters

• Completed: Boolean parameter -Either the region is defined by both extreme genes (Web Agent completes it) or the region is defined by user, who give his own boundary genes region

• Scope: this parameter is used to focus on a species subset (using TAX ID)

• "SourceURI": to select the databases to be used by the Web Agent (at the present time, the software can only use Ensembl as a data source, but adaption to other databases is possible)

• Ortholog or paralog region search: to determine type of conserved sites researched by the user

• Reverse search: to restart the process

• Range: to choose time-windows for duplication

• Distance between two genes: the orthologous clusters found are sometimes large with long non-orthologous genes gaps but can still be significantly conserved. To specify conserved region, it is possible to split the orthologous cluster if the distance between two orthologous genes is >*x *bps.

#### Phylogeny parameters

FIGENIX is Web-interfaced with configurable phylogeny parameters. Therefore, working through the CASSIOPE interface, users can configure FIGENIX at the same time, with:

• Pipeline model: FIGENIX contains several pipelines for phylogenetic reconstruction, any of which can be selected by the user. (We recommend using__CassiopePhyloM__ as it is the most up-to-date and appropriate pipeline for reliably finding orthologs and paralogs).

• Scope: to focus on a species subset or eliminate certain species from the phylogenetic analysis (which could be different from the CASSIOPE scope).

• "NoDuplicationRange": allows sequences that belong to a given taxonomic group to be considered as orthologs. For instance, in phylogenies using individual genes, human and dog sometimes appear more closely related than human and mouse, and sometimes human and mouse are closer than human and dog; to prevent a duplication node between these three species being systematically deduced, we use the NoDuplicationRange parameter by indicating [9615, 10090], which are the NCBI Taxonomic Identifiers (TaxIDs) for dog and mouse, respectively.

• Database: database used for the initial homology search (Ensembl)

• Tree of life: FIGENIX needs a reference tree of life to infer duplication and speciation nodes on tree fusions. By default, the NCBI tree of life is used, but users can set their own species tree.

## Case Study

In this section, we describe an example analysis using CASSIOPE on a test-region that was previously known to have conserved regions in vertebrate species.

### Biological data

The example region chosen contains 283 genes and is localized to chromosome 9 of the human genome from base pair 129,045,207 (ENSG00000136895: Ensembl gene identifier) to base pair 140,191,570 (ENSG00000159247). This region is an MHC-like paralogous regions [11,12]. It provides an interesting test-case as it is known to be conserved through vertebrate evolution.

Conserved regions were searched for in all of the 19 following species:

Mammals: Homo sapiens, Monodelphis domestica, Bos taurus, Mus musculus, Rattus norvegicus, Canis lupus familiaris, Equus caballus, Pongo abelii, Pan troglodytes, Macaca mulatta

Birds: Gallus gallus

Teleost fish: Takifugu rubripes, Danio rerio, Tetraodon nigroviridis, Oryzias latipes

Insects: Drosophila melanogaster, Anopheles gambiae

Nematodes:Caenorhabditis elegans

Yeast: Saccharomyces cerevisiae

### Computing features

The parameters used in this case-study are reported in Table 1.

**Table 1:** Global parameters

**CASSIOPE global parameters**	
Completed	Yes
Scope	No
Source URI	
Ortholog/paralog	Ortholog
Reverse search	Yes
Range	All

**Phylogeny parameters**	

Pipeline model	__CassiopePhylo+M__
Scope	No
NoDuplication Range	[9615, 10090]
Database	Ensembl
Tree of life	cassiope1 [see Additional file 1, figure s4]

**Cluster parameters**	

Distance between 2 genes	10,000,000 bps

CASSIOPE was run on a bi-processor machine with 2 Gb of RAM. All the agents ran on the same computer. However, as this software is based upon modular architecture, agents could be distributed over several computers.

### Output (results)

**Figure 3:**
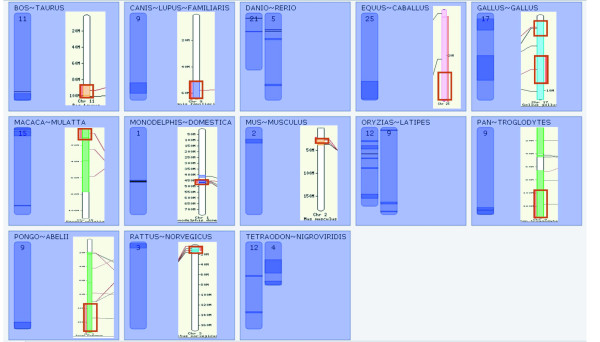
**Conserved regions from a human MHC-like region (283 genes)**. Each box represents species possessing one or several conserved regions with the start region. Regions are represented on the corresponding chromosome in the species. The right-hand-side boxes show Ensembl results (regions are delimited by red boxes), and the left-hand-side boxes show CASSIOPE results. If Ensembl results are lacking, the box is left empty. CASSIOPE found more conserved regions than Ensembl. The presence of duplication in teleost fish genomes is shown.

CASSIOPE ran for 11 days and found 1,561 conserved sites in all 19 species using reverse-search parameters. CASSIOPE launched the computation of 3,736 phylogenies via FIGENIX. Most of the execution time was spent computing phylogenetic trees, which is well-known as a time-intensive step. In order to considerably accelerate the process, CASSIOPE could also run a fast computation by using only NJ method. From the MHC-like paralogous region on human chromosome 9 (the start region of the process), 37 conserved regions were found (Figure 3). CASSIOPE then automatically computed conserved regions in all 19 species [see Additional file 1, figure s6], leading to a total of 1,561 hits. Comparing the results obtained from Ensembl data with the results obtained from the CASSIOPE process, CASSIOPE found the same regions and/or more regions for some species, as well as new conserved regions with species that were not computed in Ensembl databases. Detailed results are given in figure 3. The Ensembl results are available at: , syntenic regions link.

• For instance CASSIOPE and Ensembl found the same regions for *Bos taurus, Canis lupus familiaris, Equus caballus, Monodelphis domestica, Mus musculus, Rattus norvegicus Pan troglodytes, Pongo abelii and Gallus gallus*.

• In the case of *Macaca mulatta *CASSIOPE found one supplementary region compared to Ensembl.

• For the others species, only CASSIOPE identified conserved regions. It seems clear that the conserved regions in Ensembl are not yet fully computed.

Furthermore, CASSIOPE also statistically assessed the significance of conserved regions, providing scores.

We clearly identified a teleost-specific duplication, as was previously proposed [13]: corresponding to one region on one chromosome, we obtained several conserved regions on two chromosomes. For one chromosome in vertebrate genomes, there are two chromosomes in teleost genomes. Using the "telescope viewer", exploration of all regions highlights each gene with its detected orthologous genes, phylogenies and links to the Ensembl database (for further information on genes and access to the actual sequence, [see Additional file 1]).

## Conclusion

CASSIOPE is a reliable and flexible tool that provides access to up-to-date information based on the comparison of multiple genomes. It allows the study of conserved regions starting from a specific query genetic region. Inference of conserved gene clusters is based on phylogenetic reconstruction, which adds an historical dimension, and on statistical assessment of the significance of the inference. Finally, the built-in telescope viewer makes it easy for users to explore the results, and promptly gives an idea on the conserved regions and gene organization of each pair of species compared. CASSIOPE could be extended to other applications. In the test case reported here, CASSIOPE was only used for a global view of identified conserved regions. The CASSIOPE project is expected to give insights on genome structure and evolution and to be usefully applied in biomedical and agricultural fields (e.g., identification of QTLs or disease-gene identification). For instance, pairing information regarding the conservation of genomic regions with functional information regarding diseases could point to candidates for genes involved in pathologies.

## Authors' contributions

AL and VLR contributed equally to this work. AL, EGJD and PP designed and integrated the biological and evolutionary concepts in the system. VLR and OC carried out the software design. PG developed the first informatic system. OC and VLR have developed the final version of CASSIOPE (including structural improvements) on a software architecture proposed by PG. AL and PP wrote the manuscript. All the authors read and approved the final manuscript. Authors declare to have no competing financial or other interest in relation to this work.

## Supplementary Material

Additional file 1 **User manual and detailed results provided by CASSIOPE**. The data provided describe the CASSIOPE web page and results obtained by using CASSIOPE Click here for file
