# Supplementary material for: CASSIOPE: An expert system for conserved regions searches
Source: BMC Bioinformatics. 2009 Sep 10;10:284. doi: 10.1186/1471-2105-10-284 (PMC2756280; doi:10.1186/1471-2105-10-284)
Supplement: Additional file 1 — User manual and detailed results provided by CASSIOPE. The data provided describe the CASSIOPE web page and results obtained by using CASSIOPE [file 1471-2105-10-284-S1.doc]

**Additional file 1:**

**Figure 4:** **Cassiope1 tree of life.**

This tree corresponds to the tree of life used for duplication inference and speciation node positions. Nodes with negative numbers are virtual nodes artificially created in order to obtain a binary tree.


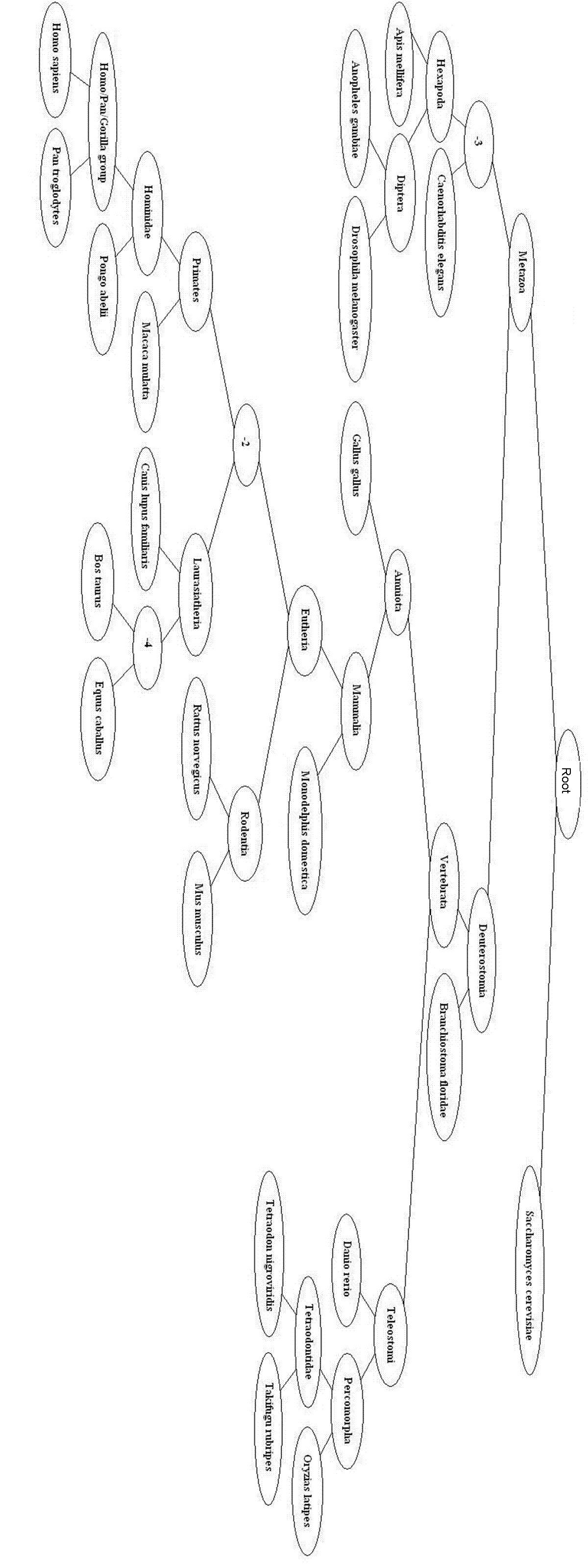


**User Manual:**

The Telescope viewer is compatible with the Firefox browser (which can be obtained freely from the Mozilla website: <http://www.mozilla-europe.org/> )

All CASSIOPE results are available at: <http://194.57.197.245/cassiopeWeb/displayCluster?clusterId=1>

Figure 5: First CASSIOPE web page – conserved regions from *Homo sapiens* to the other species


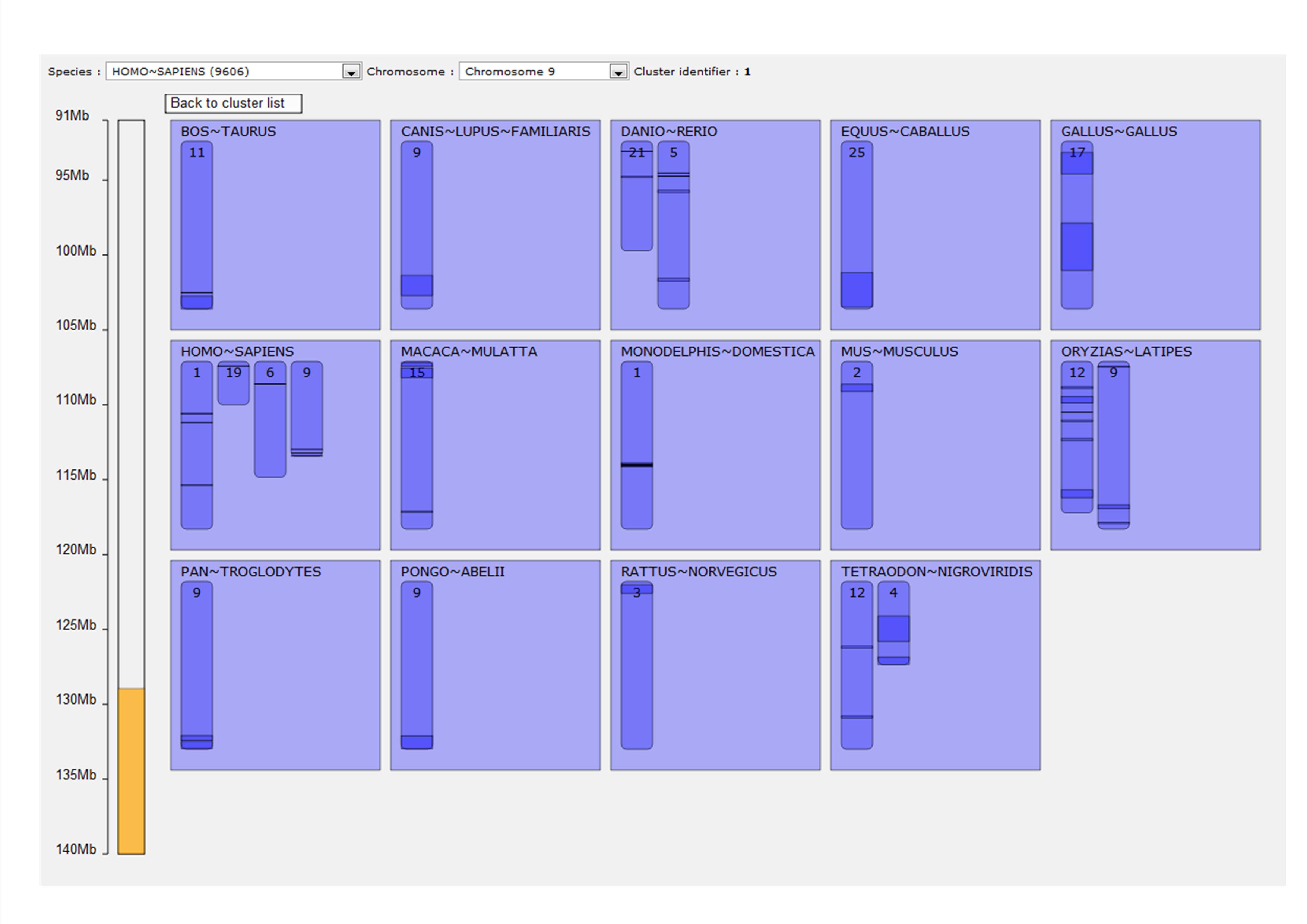


The browser displays all conserved regions from the MHC 9-like paralogous human region **(Figure 5**). Violet boxes correspond to individual species and contain all the chromosomes bearing a conserved region. A general overview of the regions is indicated in dark blue. This page depicts orthologous and paralogous conserved regions.

Clicking on a violet box, for example the *Pan troglodytes* box, shows the genes on the chromosome and displays details of the conserved region(s), as follows (**Figure 6**):


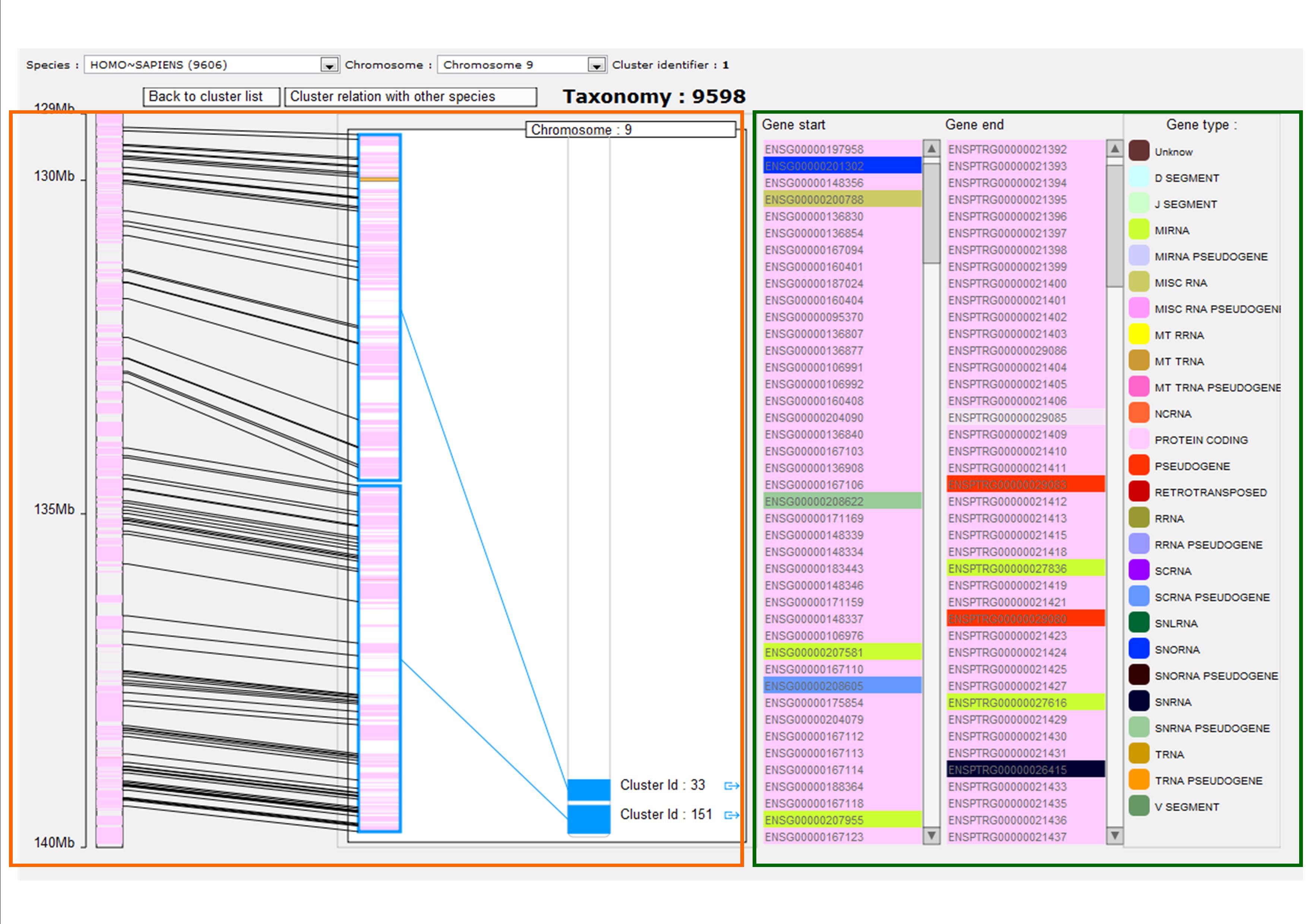
Figure 6: Conserved regions between *Homo sapiens* and *Pan troglodytes*

a)

b)

The orange box (**Figure 6a, and details on Figure 7**) contains two regions and shows links between orthologous genes. The shape on the left represents “input region” **(1)** (human region) and shapes on the right represents the corresponding conserved region **(2)** (here the Chimp). Sliding the cursor over the blue shape **(3)** or the “cluster id” label **(4)** opens a pop-up giving the conserved regions score.

Figure 7: The Orange box. Details of conserved regions between *Homo sapiens* and *Pan troglodytes*


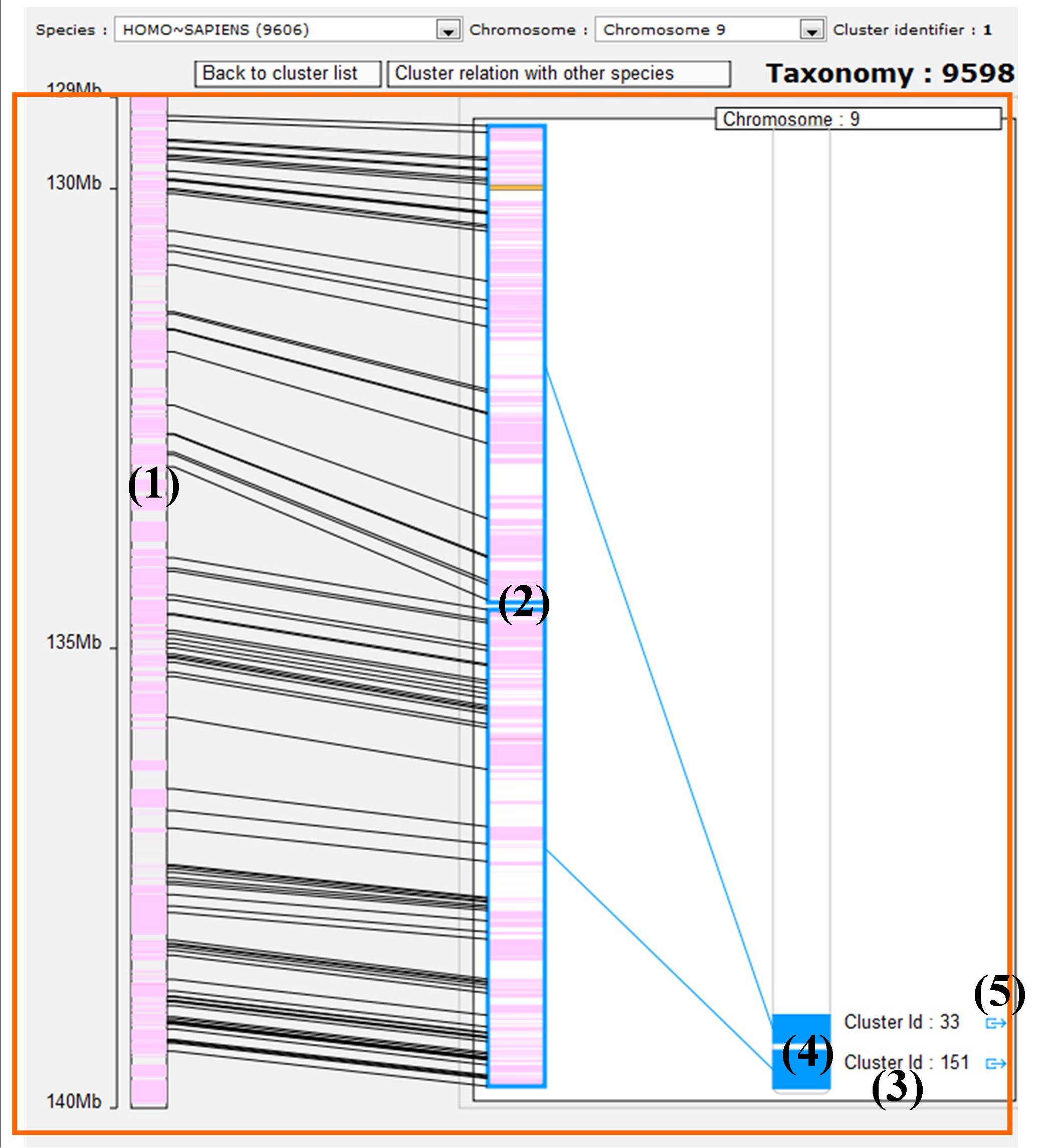


The green box (**Figure 6b**) shows all the genes present in two regions, and the color-codes system indicates type of features found (proteins in pink, pseudogenes in red, etc.). Genes for which orthology is found become yellow when the cursor moves over them. Clicking on a gene brings up a pop-up giving gene information from Ensembl databases, homologous genes, and phylogeny identifiers.

**Reverse search**

Results from a reverse search are made available by clicking the blue arrow (**Figure 7 (5)**). For example, clicking on the blue arrow of cluster 33 highlights the corresponding conserved regions of all 19 species.

Figure 8: Example of reverse search- Conserved regions from *Pan troglodytes* to the other species


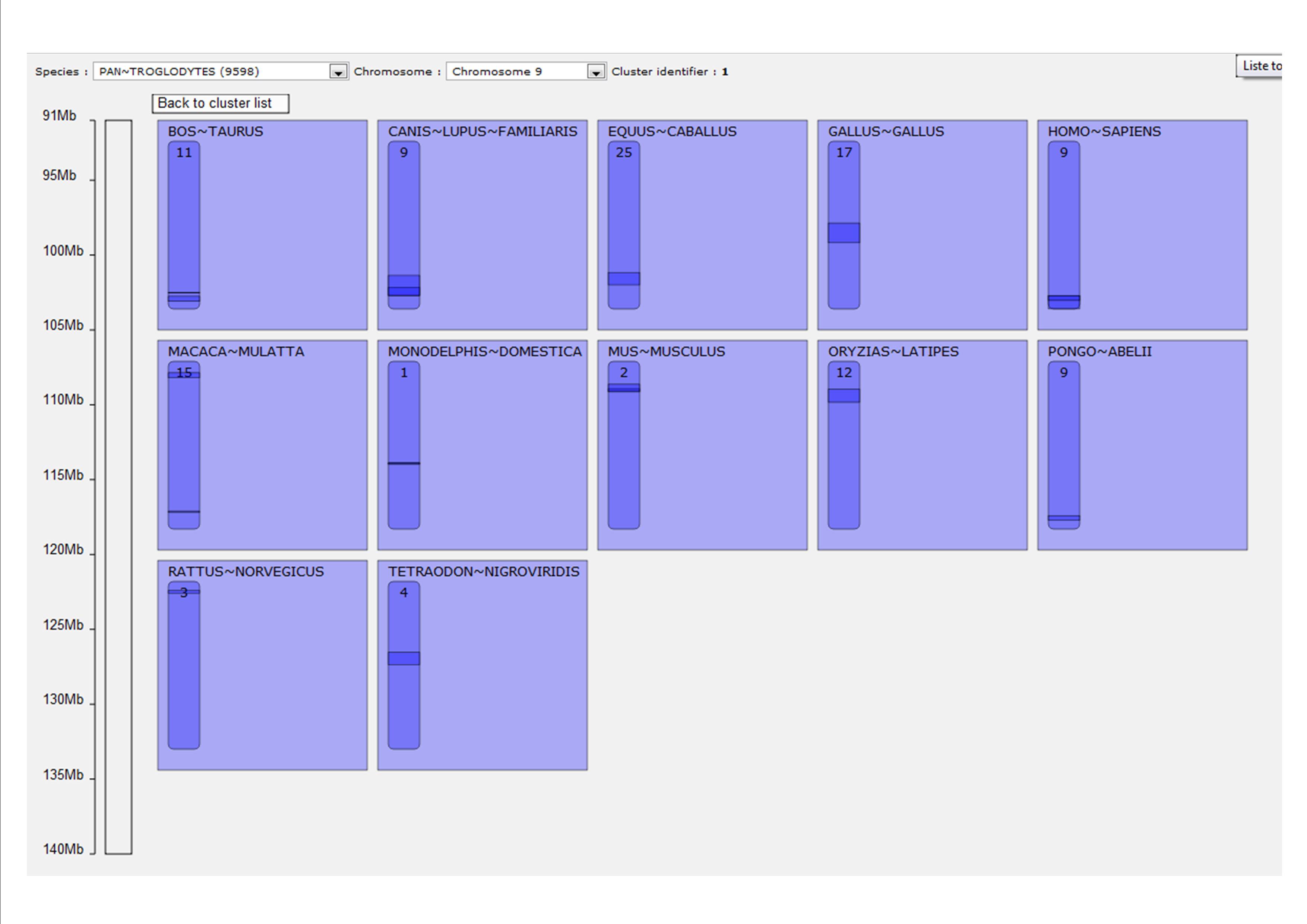


Reverse search shows the cross-species distribution of conserved regions from *Pan troglodytes* to others species. The figure shows that conserved sites with humans were found, but the human region is smaller than the human start region.
